# Physical attractiveness predicts endorsement of specific evolutionary psychology principles

**DOI:** 10.1371/journal.pone.0254725

**Published:** 2021-08-04

**Authors:** Andrew Ward, Tammy English, Mark Chin

**Affiliations:** 1 Department of Psychology, Swarthmore College, Swarthmore, PA, United States of America; 2 Department of Psychological and Brain Sciences, Washington University in St. Louis, St. Louis, MO, United States of America; 3 Harvard Graduate School of Education, Cambridge, MA, United States of America; University of Padova, ITALY

## Abstract

Evolutionary psychology has emerged as a controversial discipline, particularly with regard to its claims concerning the biological basis of sex differences in human mate preferences. Drawing on theories of motivated inference, we hypothesized that those who are most likely to be privileged by specific aspects of the theory would be most likely to support the theory. In particular, we predicted that physical attractiveness would be positively associated with endorsement of predictions of evolutionary psychology concerning mating strategies. Two studies confirmed this hypothesis. In Study 1, participants rated as higher in physical attractiveness were more likely to support specific principles of evolutionary psychology. In Study 2, a manipulation designed to boost self-perceived physical attractiveness increased endorsement of those same principles. Observer-rated physical attractiveness generally predicted individuals’ support of the theoretical principles better than did gender, political orientation, or self-esteem. Results suggest that those most likely to benefit according to certain predictions of evolutionary psychology are also those most likely to be sympathetic toward its relevant principles.

## Introduction

Evolutionary psychology, which attempts to explain the origins of psychological mechanisms by appealing to biological adaptation, has emerged as a controversial approach [1], particularly in the domain of sex differences in mate preferences [2–4]. Whereas many aspects of evolutionary psychology are undoubtedly not the subject of significant disagreement in the scientific community, some are more controversial. The evolutionary origin of putative sex differences in human behavior is among the most disputed [5]. And, among those contested sex differences, perhaps most controversial is the contention held by many evolutionary psychologists that, while men and women may pursue similar mating strategies in many contexts involving heterosexual pair-bonding, males have generally evolved to place more priority than females on physical attractiveness in a mate, whereas females have generally evolved to place more priority than males on securing a mate with the ability to provide resources [6–8]. Critics of this approach have suggested that such differences in mating strategies actually reflect differences in societal structures rather than evolutionary adaptations [9].

Given the sometimes sharp disagreements between supporters and opponents of specific aspects of evolutionary psychology [4], we sought to investigate a heretofore unexamined variable that might affect support for this particular set of controversial evolutionary psychology predictions: physical attractiveness. Drawing on theories of motivated inference [10,11], we hypothesized that endorsers of particular tenets of evolutionary psychology would be those most likely to benefit under the theory’s assumptions. In particular, physically attractive individuals, who enjoy certain mating advantages [12], were hypothesized to support specific mating-relevant predictions of evolutionary psychology.

### Present studies

In two studies, we investigated the relationship between physical attractiveness and endorsement of evolutionary psychology as it pertains to sex differences in mating behavior. In Study 1, raters evaluated the physical attractiveness of respondents who had been asked to indicate their support for evolutionary psychological principles related to female vs. male mating preferences. Such attractiveness ratings have been shown in past studies to be highly reliable, as individuals generally agree on what constitutes physically attractive features in humans [13,14], even across cultures [15]. In Study 2a, the same support was assessed after manipulating participants’ personal sense of physical attractiveness to others. Finally, in an effort to establish the discriminant validity of our predicted results, Study 2b investigated whether the effect of the attractiveness manipulation would extend to support for controversial theories other than evolutionary psychology.

## Study 1

### Materials and methods

#### Participants

Participants provided written consent for all study procedures reported in this paper, and all studies were approved by the Swarthmore College Institutional Review Board (approval code 14-15-003). A total of 84 undergraduates (43 female and 41 male, United States, college-aged, with 54 in their first year of college, 19 in their second, 8 in their third, and 3 in their fourth (mean age 18.59 years, range 17–21, with one participant not reporting)) took part in the study in exchange for partial credit toward a course requirement (and were otherwise uncompensated), which also applied to participants in the subsequent studies reported below. The mean height and weight reported by females was 65.00 inches (*SD* = 2.27) and 131.86 pounds (*SD* = 15.60), with one female reporting neither; for males, the corresponding figures were 70.54 inches (*SD* = 3.73) and 161.35 pounds (*SD* = 28.60), with one male not reporting weight. When provided with a forced-choice item, 40 participants (47.6%) indicated their political orientation as liberal, 8 as conservative (9.5%), and 35 as “middle of the road” (41.7%), with one not reporting. Finally, the average estimated family income reported by participants was, to the nearest thousand, U.S. $118,000 (7 not reporting).

*Procedure*. Participants were presented with a description of evolutionary psychology, emphasizing explanations for sex differences in mate preferences. In particular, participants were asked to read the following description:

Evolutionary psychology is a relatively new field in psychology that attempts to bring together modern evolutionary theory and psychology. In so doing, it seeks to explain present-day human behavior in terms of adaptations that were successful in evolutionary history. In other words, the past is seen as explaining present day psychological mechanisms, such as sex differences in mate preferences (i.e., what a man prefers in a mate versus what a woman prefers). For example, women are predicted to value in a mate the ability and willingness to provide social, psychological, and material resources. Men, in turn, are predicted to value those characteristics in women that provide powerful cues to reproductive value (i.e., youth and health), such as physical appearance and attractiveness.

Participants then read the following: “As you may know, evolutionary psychology has received mixed reviews among psychologists, with some strongly supporting the theory and others strongly opposed,” after which they were asked to indicate their support of evolutionary psychology on a 7-point scale (1 = *strongly disagree*; 4 = *neutral*; 7 = *strongly agree*). After completing questionnaires probing basic demographic information, participants’ permission was secured to videotape them during debriefing.

Once the data had been collected from all participants, a female and a male coder, both undergraduates who were unfamiliar with the participants, were instructed to view the relevant videotapes and rate the attractiveness of each participant using a 7-point scale (1 = *not at all attractive*; 7 = *extremely attractive*). The coders’ ratings, which were significantly correlated, *r* = .49, *p* < .001, were combined to form a mean attractiveness composite (Cronbach’s alpha = .65). Two additional raters also coded for attractiveness. Although combining all four ratings provided greater reliability to the overall attractiveness measure (Cronbach’s alpha = .77), both of the additional raters had some familiarity with a few of the participants, and thus their ratings were not employed in the resulting analysis. Importantly, the addition of their ratings did not change the pattern of findings (see below).

### Results and discussion

As predicted, participants’ attractiveness was significantly correlated with their views of evolutionary psychology, with those rated as more attractive more likely to endorse the theory, *r*(84) = .31, *p* = .004 (the addition of the ratings from the two omitted raters to the relevant composite (see above) also resulted in a significant correlation between physical attractiveness and endorsement by participants of our evolutionary psychology tenets, *r*(84) = .25, *p* = .02). Interestingly, the relationship between attractiveness and support for evolutionary psychology did not differ significantly between female (*r* = .27) and male (*r* = .31) participants, *z* < 1. Support for evolutionary psychology also did not differ significantly based on gender or political affiliation, nor was it significantly correlated with estimated family income, age, school year, height, or weight.

Participants rated as higher in physical attractiveness were more likely to agree with certain tenets of evolutionary psychology—tenets that would appear to favor those who are physically attractive [16]. Intriguingly, this relationship held equally among female vs. male participants, even though, in the provided materials, the predictions of the theory concerning physical attractiveness were explicitly mentioned only in the case of females. Of course, such a finding may reflect a version of the “matching hypothesis” [17–19], in that a theory that privileges the physical attractiveness of females could also be expected to be favored by males who consider themselves to be physically compatible with such females. In addition, endorsement of a theory that reduces behavior to certain evolved physical characteristics may be especially common among individuals of either sex who possess desirable morphological traits [15,20].

While this study supported the predicted association between attractiveness and endorsement of evolutionary psychology, its correlational nature leaves open the possibility of confounds (although measurement eliminated certain demographic variables as likely causal candidates). Accordingly, in Study 2a, we sought to manipulate physical attractiveness experimentally.

## Study 2a

### Materials and methods

#### Participants

A total of 256 (133 female and 123 male) undergraduates were randomly assigned to one of two attractiveness conditions. In the control condition, participants (*n* = 125) were asked to indicate how attractive “a typical observer” would rate their physical appearance (i.e., “How attractive would a typical observer rate your physical appearance?”; 1 = *not attractive at all*; 9 = *extremely attractive*). In the enhanced attraction condition (*n* = 131), participants were first asked to “Think of a time in which you looked your absolute best” and then to indicate the relevant “typical observer’s” ratings under those conditions. Participants were next presented with the same description of evolutionary psychology employed in Study 1, along with the item eliciting their support of the theory. In an investigation of other potentially relevant variables, participants were then asked to indicate their “general political orientation” (1 = *extremely liberal*; 4 = *middle of the road*; 7 = *extremely conservative*), along with their current mood (1 = *extremely negative*; 4 = *neutral*; 7 = *extremely positive*). Finally, they completed the Rosenberg self-esteem scale [21].

### Results and discussion

As a check of our manipulation, participants in the enhanced attraction condition responded that an observer would rate them as more attractive (*M* = 7.20, *SD* = 1.13) than did those in the control condition (*M* = 5.54, *SD* = 1.28), *t*(254) = 11.02, *p* < 001, *d* = 1.37. Consistent with our prediction, they also endorsed evolutionary psychology (*M* = 5.18, *SD* = 1.05) more than did those in the control condition (*M* = 4.85, *SD* = 1.20), *t*(254) = 2.30, *p* = .022, *d* = 0.29. This effect did not interact with participants’ gender, *F* < 1. Whereas the correlation between attractiveness and support for evolutionary psychology observed in Study 1 was conceptually replicated among control participants, *r*(125) = .18, *p* < .05, the relationship was eliminated in the enhanced attractiveness condition, *r*(131) = -.04, *ns*, possibly reflecting the reduction of variance in attractiveness and a corresponding ceiling effect in this condition. Participants in the two conditions did not differ significantly in their political orientation (*M* = 2.68, *SD* = 1.26, range: 1 (*extremely liberal*) to 6 (*somewhat conservative*)), *t*(253) = 1.17, *p* >.20, or self-esteem (*M* = 31.43, *SD* = 5.20, range: 13 to 40), *t* < 1, and although there was a trend for those in the experimental condition to report higher mood (*M*overall = 4.95, *SD* = 1.32, range: 1 to 7), *t*(253) = 1.18, *p* < .08, the basic effect was preserved when controlling for each of these three variables (all *p*s < .05). In short, asking participants to imagine themselves as physically attractive resulted in greater endorsement of a theory that, at least for females and, in terms of similarity in “mate value,” for males as well [22], privileges such attractiveness.

Of course, the possibility exists that encouraging individuals to perceive themselves as physically attractive could result in endorsement of *any* controversial hypothesis. To address this alternative, we conducted Study 2b.

## Study 2b

### Materials and methods

#### Participants

A total of 188 undergraduate participants (112 female and 76 male) took part. Participants were once again randomly assigned to the control condition or the enhanced attraction condition and underwent the same manipulation as in Study 2a. They were then exposed to descriptions of two controversial theories—one relating to psychoanalysis and one to critiques of biological approaches in psychology, with order of the two descriptions counterbalanced across participants. Specifically, participants read the following two passages:

Psychoanalysis represents a theory of personality pioneered by Sigmund Freud. According to Freud, psychological problems in an individual often have their roots in the individual’s distant past and result from an attempt to repress painful unconscious memories from childhood—memories that have to be retrieved and released before an individual can recover from mental illness.

Critics of biological approaches to psychology argue that what appear to be genetically or biologically determined behaviors are really the result of one’s culture and current social environment. For example, to the extent that sex differences in mate preference have been documented, with men putting more emphasis than women on physical attractiveness in a potential mate and women putting more emphasis than men on a potential mate’s ability to acquire material resources, those differences represent adherence to current societal norms and practices, not a genetically-determined mechanism for mate preference.

After each description, participants were asked to indicate their level of support for the relevant viewpoint (1 = *strongly disagree*; 4 = *neutral*; 7 = *strongly agree*).

### Results and discussion

Once again our manipulation check confirmed the validity of the manipulation. As in Study 2a, the two groups differed in the predicted direction in terms of how a “typical observer” would rate their physical attractiveness (enhanced attractiveness *M* = 7.20, *SD* = 1.05; control *M* = 6.34, *SD* = 1.31), *t*(186) = 4.96, *p* < .001, *d* = 0.72. However, they did not significantly differ in either their endorsement of psychoanalysis (enhanced attractiveness *M* = 3.93, *SD* = 1.56; control *M* = 3.81, *SD* = 1.55), *t* < 1, *ns*, or in their endorsement of critiques of biological approaches in psychology (enhanced attractiveness *M* = 4.41, *SD* = 1.50; control *M* = 4.70, *SD* = 1.31), *t*(186) = 1.43, *p* = .15. In short, our manipulation of reported attractiveness in the eyes of a typical other appeared limited in its (statistically significant) effect to evolutionary psychology and did not extend to two other controversial approaches in psychology.

## General discussion

Across two studies, attractiveness—either judged by raters or self-reported—was associated with a greater likelihood of endorsing evolutionary psychology. In a separate study, we ruled out the possibility that attractiveness renders individuals significantly more likely to endorse *any* controversial theory, finding that the Study 2 manipulation did not lead participants to preferentially endorse the precepts of psychoanalysis or support critiques of biological approaches in psychology.

### Comparing the results of Study 1 and Study 2

It is important to reiterate a key difference between the methods of Study 1 and Study 2. In Study 1, outside raters actually evaluated the physical attractiveness of each participant. In Study 2, by contrast, participants themselves were asked to indicate how a typical observer would rate their attractiveness. Nevertheless, the results of Study 1 and Study 2a both showed that higher ratings of attractiveness were associated with greater endorsement of particular aspects of evolutionary psychology (though, interestingly, the effect was stronger in Study 1 than in Study 2a).

To assess the overall effect across the two studies, we conducted a mini meta-analysis [23]. This analysis yielded a combined *r* of.19, a small-to-medium effect size that, using the Stouffer formula [24], was highly statistically significant, *p* < .001. In addition, it is perhaps worth noting that the findings across both studies, though differing in effect size, would seem to be highly consistent with one another, unless one were to argue that those who are rated as more physically attractive (Study 1) also somehow possess no awareness that they are seen as more attractive by others (Study 2a) or even worse, somehow think they are seen as *less* attractive by others than do those who are rated as less physically attractive. We consider such possibilities extremely remote, and we find ourselves in general agreement with Marcus and Miller [14]: “Overall, we know who is pretty or handsome, and those who are attractive know it as well” [p. 334].

### Limitations

Evolutionary psychology has been described by one critic as a field that “requires reducing people to our base instincts” [25]. Independent of the validity of such a critique, the present studies suggest that those who benefit from enhanced physical attractiveness, either as judged by others (Study 1) or themselves (Study 2), are more likely to favor aspects of evolutionary psychology that pertain to human mating.

Of course, based on reported demographic data, participants in our studies were not representative of the U.S. population as a whole, being younger, more liberal, and from a higher family income bracket than the typical U.S. citizen. They were, as well, only asked to respond to the account of evolutionary psychology that we provided to them. In order to ensure a concise stimulus paragraph, such an account was somewhat simplified, describing differences between female and male mating preferences that, while continuing to be supported by current research [26], could more properly be characterized in relative rather than absolute terms, with significant overlap between the sexes in terms of mating strategies [27].

Moreover, although our hypotheses were derived from theories of motivated inference, it is important to note that the present studies were concerned solely with documenting the existence of the relevant bias. Additional research could help explicate the underlying reasons for the favoring by physically attractive individuals of the specific predictions of evolutionary psychology that were explored in these studies. Indeed, although a motivated inference account would suggest that physically attractive individuals would favor a theory that privileges their ingroup [28], and thus they would be particularly attracted to aspects of the provided evolutionary account that highlighted the benefits of physical beauty for themselves and/or their anticipated mate, it is at least possible that such individuals were particularly drawn to other aspects of the theory, such as those privileging resource accumulation. Again, further research could help untangle these possibilities.

Although these studies included limitations and revealed modest effect sizes, the complementary approach of correlational and experimental designs bolsters the validity of the findings, which arguably can be considered substantial in the context of other plausible predictor variables [29]. Indeed, when individuals were presented with a definition of evolutionary psychology, including its application to mate preferences, observer-rated physical attractiveness best predicted support of the theory, in terms of the absolute value of the relevant correlation coefficient, *r*(84) = .31, 95% CI = [.11,.50], as compared to the next three highest contenders. These included self-esteem, *r*(125) = .22, 95% CI = [.04,.38] and political orientation (with, again, higher numbers = more conservative), *r*(125) = .21, 95% CI = [.04,.38], both assessed in the Study 2a control condition; and gender (coded as 1 = female, 2 = male), *r*(84) = .19 [-.03,.39], as assessed in Study 1, all of three which, interestingly, appeared to be much more aligned in terms of their absolute effect sizes.

## Conclusion

In summarizing a host of published studies, Ditto et al. [30] argued that “[p]eople are less skeptical consumers of information that they want to believe than of information that they do not want to believe…and this pattern is as evident in the political realm as it is in other realms of life that evoke strong emotions, preferences and social allegiances” [p. 11]. As an account that can provoke strong “emotions and preferences,” evolutionary psychology, particularly as applied to human mating preferences, would seem to represent an ideal target for such responses from those who are more privileged versus less privileged by the theory.
